# Environmental vulnerability of the global ocean epipelagic plankton community interactome

**DOI:** 10.1126/sciadv.abg1921

**Published:** 2021-08-27

**Authors:** Samuel Chaffron, Erwan Delage, Marko Budinich, Damien Vintache, Nicolas Henry, Charlotte Nef, Mathieu Ardyna, Ahmed A. Zayed, Pedro C. Junger, Pierre E. Galand, Connie Lovejoy, Alison E. Murray, Hugo Sarmento, Tara Oceans coordinators, Silvia G. Acinas, Marcel Babin, Daniele Iudicone, Olivier Jaillon, Eric Karsenti, Patrick Wincker, Lee Karp-Boss, Matthew B. Sullivan, Chris Bowler, Colomban de Vargas, Damien Eveillard

**Affiliations:** 1 Université de Nantes, CNRS UMR 6004, LS2N, F-44000 Nantes, France.; 2 Research Federation for the study of Global Ocean Systems Ecology and Evolution, FR2022/Tara Oceans, Paris, France.; 3 Sorbonne Université, CNRS, Laboratoire Adaptation et Diversité en Milieu Marin, Station Biologique de Roscoff, 29680 Roscoff, France.; 4 Institut de Biologie de l’École Normale Supérieure (IBENS), École Normale Supérieure, CNRS, INSERM, PSL Université Paris, 75005 Paris, France.; 5 Department of Earth System Science, Stanford University, Stanford, CA 94305, USA.; 6 Sorbonne Université, CNRS, Laboratoire d'Océanographie de Villefranche, LOV, F-06230, Villefranche-sur-Mer, Paris, France.; 7 Department of Microbiology, Ohio State University, Columbus, OH 43210, USA.; 8 Department of Hydrobiology, Universidade Federal de São Carlos (UFSCar), Rodovia Washington Luiz, 13565-905 São Carlos, SP, Brazil.; 9 Sorbonne Université, CNRS, Laboratoire d’Ecogéochimie des Environnements Benthiques, LECOB, Banyuls-sur-Mer, 66500 Paris, France.; 10 Département de biologie, Faculté des sciences et Institut de biologie intégrative et des systèmes (IBIS) 1030, ave de la Médecine, Université Laval, Québec, QC, Canada.; 11 Division of Earth and Ecosystem Science, Desert Research Institute, Reno, NV 89512, USA.; 12 Department of Marine Biology and Oceanography, Institut de Ciències del Mar (CSIC), Barcelona 08003, Spain.; 13 Takuvik International Research Laboratory, Université Laval and CNRS, Québec, QC, Canada.; 14 Stazione Zoologica Anton Dohrn, Villa Comunale, Naples 80121, Italy.; 15 Génomique Métabolique, Genoscope, Institut François Jacob, CEA, CNRS, Université Evry, Université Paris-Saclay, Evry, 91057 Paris, France.; 16 School of Marine Sciences, University of Maine, Orono, ME, USA.; 17 Department of Civil, Environmental and Geodetic Engineering, Ohio State University, Columbus, OH 43210, USA.

## Abstract

Marine plankton form complex communities of interacting organisms at the base of the food web, which sustain oceanic biogeochemical cycles and help regulate climate. Although global surveys are starting to reveal ecological drivers underlying planktonic community structure and predicted climate change responses, it is unclear how community-scale species interactions will be affected by climate change. Here, we leveraged *Tara* Oceans sampling to infer a global ocean cross-domain plankton co-occurrence network—the community interactome—and used niche modeling to assess its vulnerabilities to environmental change. Globally, this revealed a plankton interactome self-organized latitudinally into marine biomes (Trades, Westerlies, Polar) and more connected poleward. Integrated niche modeling revealed biome-specific community interactome responses to environmental change and forecasted the most affected lineages for each community. These results provide baseline approaches to assess community structure and organismal interactions under climate scenarios while identifying plausible plankton bioindicators for ocean monitoring of climate change.

## Teaser

A global photic-ocean plankton ecological network predicts distinct vulnerabilities to environmental change across marine biomes.

## INTRODUCTION

Marine plankton and associated processes are at the core of global biogeochemical cycles, shaping ecosystem structure and influencing climate regulation (*1*). While global biodiversity maps for viruses, prokaryotes, and microbial eukaryotes are beginning to emerge (*2*–*4*), identifying and understanding the complex network of interactions between these organisms and their environment is in its infancy (*5*). These interactions are critical to establish the ecosystem trophic links that underpin biogeochemical cycles and feedbacks that drive climate regulation and response (*6*, *7*). While abiotic factors, such as temperature, can explain a large fraction of microbial community composition in the global ocean (*8*), biotic interactions can differentially shape ecosystem diversity (*9*) and can even influence the adaptation to new environments (*10*). Thus, determining how plankton ecological interactions are structured and affected by environmental change remains a notable challenge.

Large-scale holistic marine ecosystem sampling facilitates conceptualization of plankton community interactomes as co-occurrence networks that are useful to model the complex community structure of ecological associations (*11*, *12*). These networks have enabled the detection of communities assembled through niche overlap across biomes (*13*) and also the prediction of putative interactions such as parasitism or symbioses (*14*). Likewise, plankton co-occurrence networks have been instrumental in detecting interrelated changes in community structure from surface to depth (*15*), as well as in identifying specific communities of key lineages (e.g., *Synechococcus*, its phages, and Collodaria) associated with global open ocean processes such as carbon export (*16*). Community interactomes are also useful to identify central, highly connected lineages that may play significant ecological roles and confer stability to the community (*17*). These central lineages can correspond to keystone taxa that are good indicators of community shifts (*18*). Understanding the mechanisms affecting these central taxa may help us to predict responses of microbiome structure and functioning to perturbations (*19*).

While community interactomes inferred from global-scale samplings summarize well the complexity and potential interactions within microbial assemblages (*12*), they usually do not reflect dynamic processes shaping the observed system, as measured by longitudinal high-frequency sampling (*20*). Thus, alternative strategies need to be developed to capture ecosystem dynamics and responses from spatial samplings. Plankton species display various ecological and evolutionary responses to global environmental change (*21*, *22*). Within marine ecosystems, the interplay between species ecological niche and climate change can induce abrupt community shifts, which may lead to long-term reconfiguration of marine metazoan communities or biodiversity rearrangements (*23*, *24*). Recently, environmental drivers of ocean plankton diversity were inferred from *Tara* Oceans data and used to predict the effects of severe warming on surface ocean biodiversity (*3*). While species niche distribution models combined with climate models are useful to project fine-scale future distributions of species (*25*), species interactions are generally not included in these models (*26*), certainly due to our lack of knowledge about organismal interactions. Nevertheless, plankton network topological metrics can capture emergent properties (e.g., connectivity) relating to ecological characteristics of the community (*27*), which can serve as proxies of ecosystem and community-level resilience (*28*). Given that biotic interactions can influence species distributions at macroecological scales (*29*) and that climate change may cause trophic cascading effects on plankton community structure by directly affecting the top and bottom of marine food webs (*30*), ecological interactions need to be considered for assessing plankton community stability under climate change scenarios.

Here, we connected ecological and climate modeling by combining network analyses (*31*) with species niche models (*32*) into a computational framework for predicting ecosystem-scale vulnerabilities to environmental change. By leveraging *Tara* Oceans data from all major oceanic provinces, including the Arctic Ocean, we inferred a comprehensive global ocean cross-domain plankton co-occurrence network from sequencing data. We built statistical niche models to predict realized niches of planktonic taxa, across kingdoms, and from pole to pole. These were then mapped onto the network and used to evaluate both local and global robustness of plankton community structures to simulated environmental changes. In addition, we integrated climate model projections [CMIP6 model (*33*) scenario SSP2-4.5] for predicting affected proportions of plankton taxonomic groups, for which we considered environmental ranges corresponding to global mean anomalies projected for the end of the century. Noticeable efforts have used the ecological niche concept to identify open ocean physical conditions governing phytoplankton biogeography (*34*) and also to better formalize central biogeochemical processes through the definition of key plankton functional types (*35*). The niche representation of planktonic diversity affords a more effective integration of abiotic and biotic constraints to better predict perturbations of primary productivity under climate change scenarios (*36*).

## RESULTS AND DISCUSSION

### A cross-kingdom plankton interactome from pole to pole

**Fig. 1:**
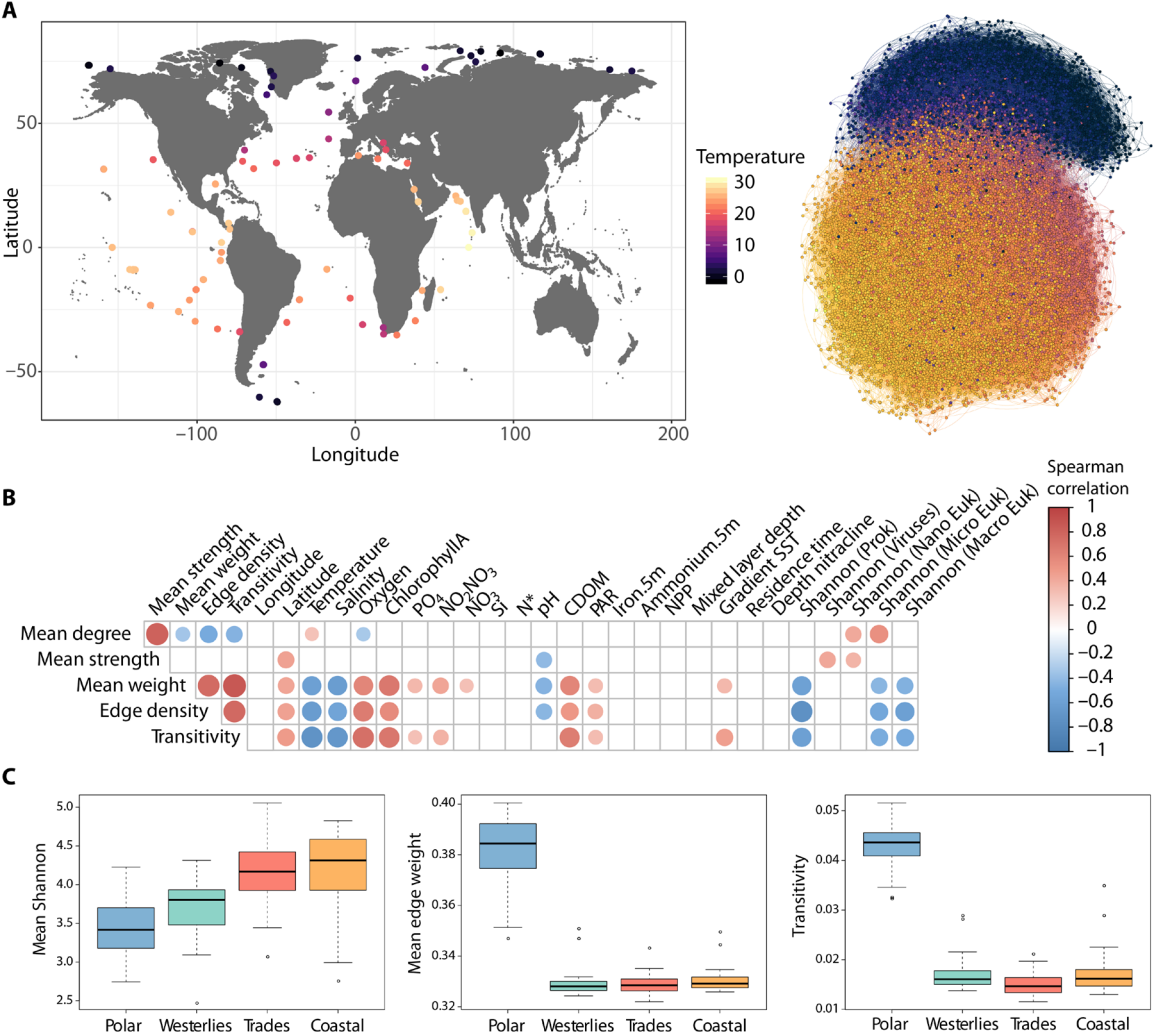
Abiotic factors shape the pole-to-pole cross-domain plankton interactome structure. (**A**) The *Tara* Oceans circumnavigation (2009–2013) included a comprehensive metabarcoding and metagenomics sampling along with physicochemical parameter measurements covering a wide pole-to-pole latitudinal gradient of temperature. The GPI covers the three domains of life including eukaryotes, bacteria, and archaea and is highly structured along the latitudinal gradient of temperature from the equator to the poles. It counts 20,810 nodes (and 86,026 edges) colored according to their optimum niche temperature. (**B**) The plankton interactome topology is significantly associated to diversity, temperature, salinity, light (PAR, photosynthetically available radiation), nutrient concentrations, and pH (Spearman correlations FDR < 0.01, empty boxes correspond to nonsignificant correlations). (**C**) The polar interactome displays stronger associations (mean edge weight) and clustering coefficients (transitivity) compared to other biomes (Dunn’s test, FDR < 0.05) despite its overall lower diversity.

To reconstruct a global marine plankton co-occurrence network across kingdoms of life, we analyzed data from 115 stations from the *Tara* Oceans expeditions (2009–2013) covering several organismal size fractions and all major oceanic provinces (*37*) across an extensive latitudinal temperature gradient from pole to pole (Fig. 1A). Using a dedicated probabilistic learning algorithm (*38*) (see Materials and Methods), we predicted ecological interactions between plankton taxa from compositional abundances inferred from sequencing data. The resulting integrated species association network [referred to as the Global Ocean Plankton Interactome (GPI)] counts a total of 20,810 nodes corresponding to operational taxonomic units (OTUs) and 86,026 edges corresponding to potential biotic interactions (Fig. 1A). In comparison to a previous plankton interactome generated from *Tara* Oceans data (*14*), GPI doubled the number of recovered known interactions from the literature (see Supplementary Text). A vast majority of positive associations (98.5%) were predicted, probably underlying a prevalent role for biotic interactions in shaping marine plankton communities (*14*). Very few direct associations between OTUs and environmental parameters were detected (*n* = 325; see Supplementary Text). However, by estimating robust ecological optima (niche value) and tolerance ranges (realized niche widths) (*39*) for each OTU and environmental parameter (see Materials and Methods and table S3), we observed a strong influence of temperature in structuring predicted interactions (Fig. 1A). The GPI displayed a very high temperature optima assortativity coefficient (AC_t_ = 0.87), which quantifies the tendency of nodes being connected to similar nodes (here with similar temperature niche optima) in a network. Thus, it confirms that the latitudinal temperature gradient indirectly shapes the GPI (*3*) and demonstrates the substantial effect of both environmental forcing and habitat filtering in structuring marine plankton communities at the global scale.

### Abiotic factors differentially shape the plankton interactome structure

To further investigate the influence of abiotic factors in shaping the GPI structure, we extracted local subnetworks corresponding to potential interactomes at each sampling site and computed graph topological metrics. These local metrics (see Supplementary Text for a detailed description) were then correlated to environmental parameters (see Materials and Methods). The relationship between species diversity, network complexity, and ecological stability is a major topic of interest in ecology, and diverse relationships between complexity and stability have been observed in mutualistic networks (*40*). Here, we generally assumed that a higher connectivity would be associated to higher ecological stability and robustness, with highly connected communities being more persistent and resilient. Globally, the GPI network connectivity assessed by these metrics was negatively associated with temperature and salinity (Fig. 1B and Supplementary Text), pointing toward their potential impact in altering the structure of predicted interactions (*41*). We also observed a differential association between temperature and interactome connectivity in polar (fig. S1A; negative association trend with mean strength, Spearman rho = −0.37, *P* = 9.4 × 10^−2^) versus nonpolar regions (significant positive association with mean strength, Spearman rho = 0.43, *P* = 1.8 × 10^−4^). This difference may be linked to the observation that community turnover, which dominates in polar versus nonpolar prokaryotic communities (*4*), is accompanied by stronger biotic dependencies between species. It also suggests a potential role for temperature in reducing polar community connectivity in response to ocean warming, which we modeled and discuss below.

Given the observed differential association between temperature and community structure along the latitudinal axis, we compared local interactome topological metrics across biomes (Fig. 1C). The network stability (mean weight) and connectivity (transitivity) were significantly higher for the polar biome compared to other marine biomes [Dunn’s test, false discovery rate (FDR) < 0.05 for all tests] and were associated with a lower mean (cross-domains) species diversity. This higher connectivity of the polar interactome is intriguing and suggests a more prevalent role of biotic interactions in structuring less diverse plankton communities in the extreme polar environment. A potential explanation for this higher connectivity may be the high abundance of ubiquitous diatoms in polar regions, which have been reported as selective segregators of global ocean plankton communities, displaying a very high proportion of associations with other organisms in a previous interactome (*42*). In addition, a potential higher prevalence of mixotrophic plankton in the Arctic (e.g., mixotrophic flagellates and ciliates) may be responsible for enhancing the connectivity of polar trophic food webs (*43*). Alternatively, this higher connectivity may also reflect the more complex food web structures that vary across polar regions, although they are characterized by specific pathways of energy flow dominated by a small number of species (*44*). Our observation may also be linked to the influence of temperature in globally shaping physiological and ecological traits across levels of organization. The overall increase in traits performance for prey (relative to predators) at lower temperature (*45*) could result in a stronger predator-prey arms race and thus a potential higher connectivity in polar regions.

The lower plankton richness and diversity observed in polar ecosystems have also been linked to maximal species turnover and environmental variability (*46*), which may translate into a higher detectable connectivity between distinct species in the polar interactome. Here, we hypothesized that a higher species turnover and/or a higher environmental heterogeneity (despite lower diversity) may facilitate the detection of associations between organisms and thus explain the higher connectivity observed in the polar interactome. Nevertheless, the environmental heterogeneity of polar ecosystems may also result in higher heterogenous selection and community turnover, thus further increasing network connectivity. As recently proposed for a fluvial river system, environmental heterogeneity may determine the ecological processes assembling bacterial metacommunities (*47*).

### Biome-specific communities emerge from the plankton interactome

**Fig. 2:**
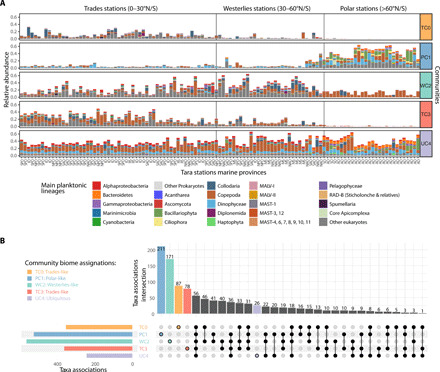
Biome-specific communities and associations emerge from the plankton interactome. (**A**) The GPI can be decomposed into five communities that are preferentially observed in specific marine biomes: Communities TC0 and TC3 are Trades-like, community WC2 is Westerlies-like, community PC1 is Polar-like, and community UC4 is ubiquitous. Distinct main plankton lineage compositions are observed in each community along the latitudinal axis (stations are ordered by absolute latitude), disrespect of the ocean region. SPO, South Pacific Ocean; NPO, North Pacific Ocean; SAO, South Atlantic Ocean; NAO, North Atlantic Ocean; IO, Indian Ocean; RS, Red Sea; MS, Mediterranean Sea; SO, Southern Ocean; AO, Arctic Ocean. (**B**) Most plankton associations between main plankton lineages are community-specific, with communities WC2 (Westerlies-like) and PC1 (Polar-like) displaying the highest number of discriminant associations, while community UC4 displays fewer ubiquitous associations. Shared associations between communities are indicated with black-filled circles and connecting lines.

To further our understanding of the role of temperature in shaping the interactome structure along the latitudinal axis, we used an unsupervised approach to delineate network communities and test their association with specific biomes. Using a deterministic community detection algorithm (see Materials and Methods), five communities emerged from the GPI, which were enriched in OTUs assigned to specific biomes, and displayed distinct predicted biotic associations (Fig. 2). Through comparison of community abundance profiles, these five communities were preferentially observed in specific biomes (Fig. 2A and table S4). GPI communities 0 and 3 (TC0 and TC3) occurred preferentially in Trades stations, community 2 (WC2) prevailed in Westerlies stations, while community 1 (PC1) emerged in Polar stations. Community 4 (UC4) was more abundant in Polar stations but displayed a clear ubiquitous distribution. This unsupervised approach to community detection demonstrates that the GPI is self-organized across marine biomes and that it captures the biogeography of cross-domain plankton associations. It also indicates that Longhurst’s primary biome partitioning (*48*), which is based on chlorophyll and phenology, is also biologically meaningful for planktonic associations across plankton domains and size spectra.

All GPI communities (with the exception of the ubiquitous UC4) displayed mostly exclusive associations, even at the high taxonomic level of the main planktonic lineages considered (Fig. 2B). All GPI communities differed in their associations (fig. S3), which were enriched between distinctive taxa (table S6). Most prevalent associations in communities TC0, TC3, and WC2 (see Supplementary Text) included radiolarians (e.g., Spumellaria, Acantharea, and Collodaria) and Dinophyceae, detected in associations with parasitic organisms [e.g., marine alveolates (MALV); see Supplementary Text for details]. Both PC1 (Polar-like) and UC4 (ubiquitous) communities formed two distinct systems as compared to TC0, TC3, and WC2 with respect to co-occurring lineages (fig. S3).

The PC1 community displayed a significantly lower contribution of MALV and was particularly enriched in Bacillariophyta (diatoms) associations not only with several eukaryotic lineages, including Ciliophora, Cryomonadida, Choanoflagellatea, and Mamiellophyceae, but also with bacterial lineages such as Bacteroidetes and Gammaproteobacteria, suggesting widespread diatom-bacteria interactions (*49*) in polar ecosystems. Bacillariophyta-Cryomonadida associations may correspond to ecologically important interactions in sea ice–influenced waters. Several Cryomonadida in cold waters can feed on diatoms, and some *Cryothecomonas* spp. are diatom parasitoids (*50*). In particular, we observed enriched associations between Bacillariophyta, Ciliophora, and Cercozoa in the PC1 community (fig. S4A and Supplementary Text). These results imply a broader range of potential interactions than previously thought between these groups and, in particular, regarding the diversity of diatom genera that can be infected by Cryomonadida and predated by *Strombidium* ciliates.

The UC4 community was significantly enriched in associations involving heterotrophic bacterial lineages (Alphaproteobacteria, Gammaproteobacteria, and Bacteroidetes) between themselves and with major phytoplankton taxa such as Dinophyceae, Haptophyta, and Bacillariophyta, among the most abundant photosynthetic eukaryotes (*51*). Notable UC4 overrepresented associations (table S6) included Haptophyta with MAST (marine stramenopiles) and MALV lineages, emphasizing the promiscuous nature of MALV parasitic interactions not only in tropical and temperate ecosystems (*14*) but also in polar regions. Several cross-domain associations were enriched in UC4, such as Bacillariophyta and Dinophyceae with Bacteroidetes, and Copepoda with Alphaproteobacteria, revealing the pervasive role of phytoplankton- and zooplankton-bacteria ecological interactions (*52*) shaping the plankton microbiome from pole to pole. UC4-enriched associations also involved the most abundant bacterial taxa in the oceans, such as SAR11 (and SAR116), SAR86, Rhodobacterales, and Flavobacteria (*53*). This pattern (fig. S4B) may result from the competition for limiting resources in which the same organic sources are consumed by these bacteria; this is the case, for instance, for dimethylsulfoniopropionate (DMSP), a chemical signalization molecule involved in microbial interactions in the ocean, as well as a carbon and sulfur source preferentially consumed by SAR11, SAR86 (*54*), and Rhodobacterales (*55*). It has been shown that the degradation of dissolved organic matter is not dominated by one specific phylogenetic group but rather that different bacterioplankton have specialized in the degradation of different compounds (*56*). Thus, the shared preference for these resources is not sufficient to explain the association patterns of these microbial taxa at the global scale. One interesting concept known as “division of labor” allows community microbes to survive with minimal energy resources by combining their metabolic activities, either by direct metabolite exchange or by syntrophy, where the receiver benefits from the opportunistic consumption of a metabolic by-product it is not able to produce, thus increasing the fitness of the whole community (*57*). Bacterioplankton associations enriched in UC4 emphasize the nonrandom patterns of prokaryotic interactions, which appear to be shaped by a complex combination of competition and cooperation, from pole to pole. Core associations detected across all GPI communities were also identified (*n* = 56; Fig. 2B) and reflected strong dependencies between clades that have coadapted to specific environmental conditions encountered in each biome. These core associations were dominated by MAST and MALV lineages, underlying their broad biogeography (*58*), and very successful adaptation from pole to pole, through grazing and parasitism, respectively.

Communities emerging from the GPI underline niche differentiation by biome and imply that community-specific ecologically central species may be identified. To identify species whose impacts appear to be particularly important compared to their abundances, we computed the integrative general keystone index for each GPI community (*59*). Focusing on the ubiquitous UC4 community, the top 10 OTUs delineated by the index (table S7) included Eukarya (*n* = 6), among which several Copepoda (Cyclopoida, *Corycaeus* sp.) and Dinophyceae (Phalacroma, *HM581743* sp.) taxa. It also included bacterial OTUs (*n* = 4) belonging to AEGEAN-169, NS5 marine group, *Polaribacter*, and SAR116 lineages. The AEGEAN-169 group was previously shown to be abundant and ecologically important at the San Pedro Ocean Time Series (SPOT) station (*60*). *Polaribacter* environmental genomes were recently shown to be prevalent and active in the euphotic zone at both poles (*61*).

Although each GPI community was more abundant in a given biome, their occurrence goes beyond these partitions, which probably reflects the importance of physical processes (e.g., advection by ocean currents) influencing their distribution through dispersal (*62*). This is also reflected by the biogeography of the WC2 community (Westerlies-like) and especially UC4 that is ubiquitous and appears to interface with other communities. The broader biogeography of these associations reflects the interconnected evolutionary history of phytoplankton- and zooplankton-bacteria ecological interactions and their pervasive role in influencing fundamental processes such as primary production, nutrient regeneration, and biogeochemical cycling (*52*) not only in low-nutrient regions of the ocean but also from pole to pole.

### Community-specific vulnerabilities to environmental change

Given that the GPI captured the global biogeography of cross-domain plankton associations, we sought to investigate the potential influence of environmental change on community stability across biomes. Unlike previous studies that mapped global biodiversity and investigated ecological drivers, we used the GPI as a basis to develop a novel computational framework integrating OTU niche inference and community network analyses to assess how plankton communities and lineages may be affected under environmental change. First, for each OTU, we calculated the ecological optimum and tolerance range for a selection of environmental parameters including salinity, nutrient concentrations (NO_2+_NO_3_, PO_4_), pH, and temperature. These abiotic factors are projected to change significantly under ongoing climate change scenarios (*36*). For the temperature niche, we observed smaller OTU tolerance ranges toward the poles and the equator (fig. S5), which supports the general assumption of higher environmental stability and narrower temperature ecological niches in both Polar and Trades biomes compared to the Westerlies (*48*). The environmental optima and tolerance ranges of OTUs inform us about the realized ecological niches of the taxa they represent and their potential sensitivity to environmental variations. OTUs from taxa with narrower tolerance ranges (i.e., specialists) are more likely to be affected by environmental changes, while OTUs from taxa with larger tolerance ranges (i.e., generalists) are more likely to be less sensitive to environmental changes. On the basis of this general assumption, we then simulated the effect of environmental changes on plankton interactome stability. Specifically, we perturbed GPI by progressively removing nodes ranked by their environmental tolerance ranges, from the narrower to the wider, for each parameter. We also attacked GPI’s nodes by their degree (i.e., from the most connected to the least connected nodes) to simulate the potentially most damaging perturbation of the network and repeated random attacks to obtain a random expectation reference. The GPI perturbations were systematically performed at the global scale to study both global and community-specific impacts of these attacks on the stability of the network (see Materials and Methods).

**Fig. 3:**
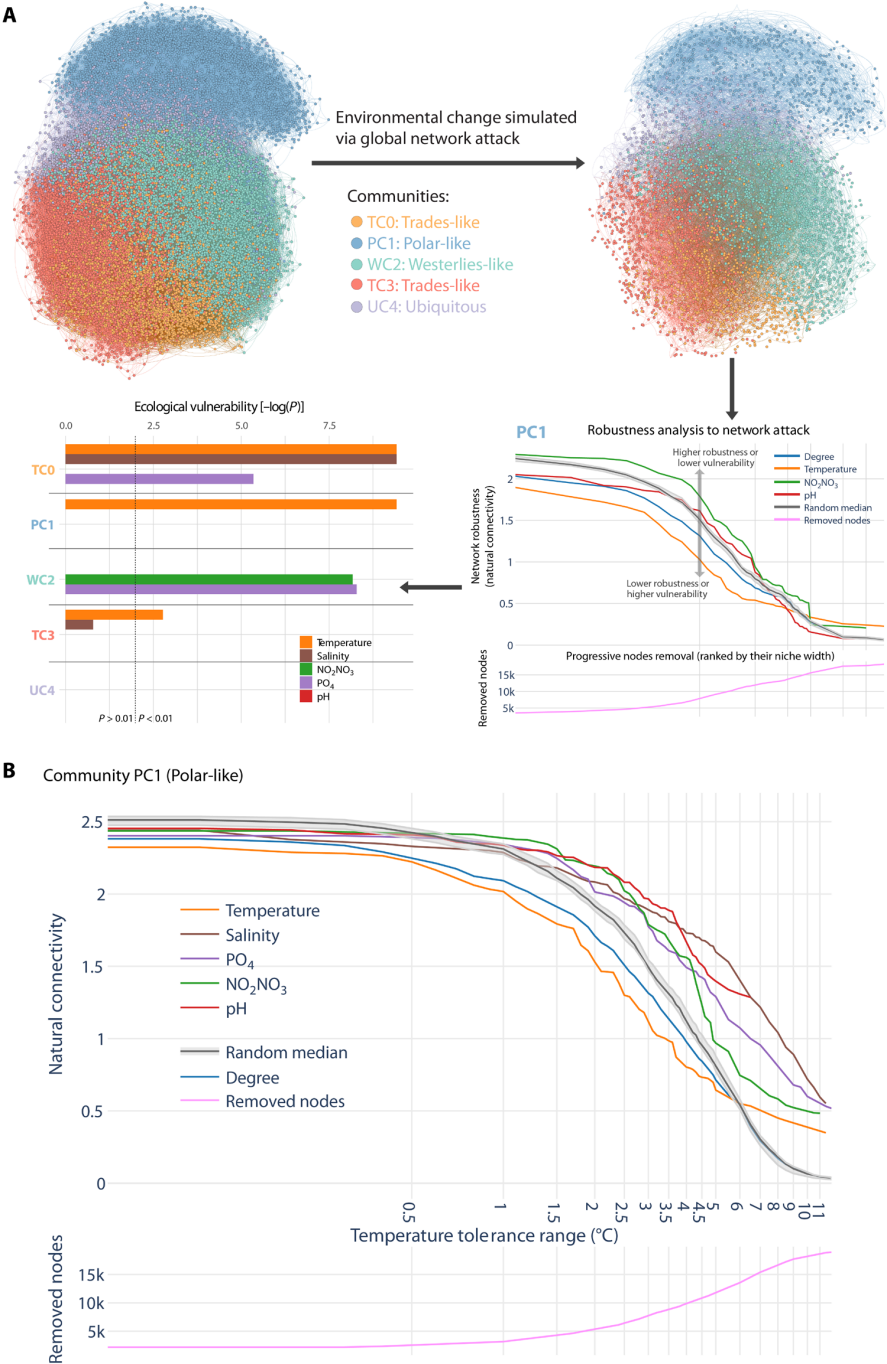
Predicting ecological vulnerabilities via network-based simulations. (**A**) Environmental change simulations are performed through tolerance range perturbations, which is progressively removing nodes of the GPI ranked by their environmental niche width (from smaller to larger), to predict ecological vulnerabilities of GPI communities. Significant vulnerabilities to environmental changes were determined by comparing distributions of the network natural connectivity (a graph robustness measure) evolution for each abiotic factor, as compared to a random perturbation. The ecological vulnerability of each GPI community was then quantified by the statistical significance [−log(*P*)]. GPI communities TC0, TC3 (Trades-like), and PC1 (Polar) were predicted vulnerable to temperature change, while community WC2 (Westerlies-like) was predicted vulnerable to nutrient concentration variations. (**B**) The polar community (PC1) is predicted to be particularly vulnerable to temperature variations (Wilcoxon rank test, *P* = 3.8 × 10^−10^).

In response to in silico environmental perturbations, we observed an overall global robustness of the network (fig. S6). However, at the local scale, we found evidence for differential effects of specific abiotic factors on GPI communities (Fig. 3A). While the UC4 (ubiquitous) community was found to be the least sensitive to simulated environmental changes (table S8; *P* > 1 × 10^−2^), community TC0 (Trades-like) displayed significant vulnerabilities (fig. S7A) to temperature (Wilcoxon rank test, *P* = 3.8 × 10^−10^), salinity (*P* = 3.8 × 10^−10^), and PO_4_ (*P* = 4.5 × 10^−6^), as compared to random attacks. The TC3 community (Trades-like) also displayed a significant vulnerability to temperature (*P* = 1.7 × 10^−3^). The WC2 community (Westerlies-like) was predicted as being the most vulnerable (fig. S7B) to nutrient concentration changes (NO_2_ + NO_3_, *P* = 6.7 × 10^−9^; PO_4_, *P* = 5.2 × 10^−9^), while the PC1 community (Polar-like) displayed a clear vulnerability (Fig. 3B) to temperature (*P* = 3.8 × 10^−10^). These distinct predicted sensitivities of GPI communities imply that taxa represented by central, most connected OTUs display lower environmental tolerance ranges for distinct abiotic factors in each community. Thus, these findings suggest that the plankton interactome will be affected differently by environmental change in specific ecological marine regions, which are themselves predicted to be affected differently by warming and nutrient distributions (*63*). Both Trades communities (TC0 and TC3) appeared to be more sensitive to temperature and, to a lesser extent, to salinity, which are both currently increasing in tropical ocean regions (*64*). On the other hand, the Westerlies community (WC2) appeared to be more vulnerable to nutrient concentration variations, which is a coherent scenario with climate change projections (*36*). These predictions also confirm the vulnerability of the Polar community (PC1) to temperature changes that are currently occurring with the rapid warming of the Arctic over recent decades and that is projected to be amplified (*65*).

### Plankton lineages potentially most affected by environmental change

**Fig. 4:**
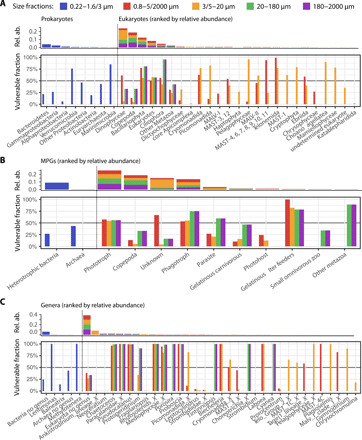
Polar marine plankton lineages and groups predicted to be most vulnerable to temperature change. (**A**) Environmental tolerance range perturbations of the GPI predicted polar marine plankton lineages (community PC1) potentially most affected by temperature variations. (**B**) Grouping these lineages into MPGs predicted associated functions potentially most affected by temperature variations in the polar ecosystem. (**C**) Genera most impacted by temperature variations are also identified and are potential species indicators of ocean warming in the polar ecosystem. In all panels, the fraction of lineages, MPGs, and genera (from 1 for most affected to 0 for not affected) predicted to be affected by temperature variations are depicted within each size fraction. Plankton lineages (prokaryotes and eukaryotes), MPGs, and genera are ordered according to the cumulative mean relative abundance of the corresponding OTUs across size fractions (note that these relative abundances are not directly comparable between size fractions).

By combining environmental tolerance range inference with network stability analyses, plankton communities most affected by environmental perturbations were predicted, as well as vulnerabilities of the respective plankton taxa and marine plankton groups (MPGs; see Materials and Methods). For temperature vulnerability predictions, we considered relatively abundant OTUs displaying a temperature niche width smaller than 2.1°C, which corresponds to the global mean sea surface temperature anomaly projected for the end of the century by the CMIP6 model scenario SSP2-4.5 (*33*). Marine plankton vulnerabilities to temperature, salinity, and nutrient concentration changes were predicted for communities TC0 and WC2 (see Supplementary Text). Focusing on the PC1 polar community, which appeared to be the most sensitive to temperature change, we identified specific plankton lineages from all domains of life predicted to be affected (Fig. 4A). The bacterial phyla Verrucomicrobia and Marinimicrobia were found most sensitive with a vulnerable fraction above 50%. Verrucomicrobia lineages are poorly characterized but are ubiquitous in the ocean and may be essential for the biogeochemical cycling of carbon (*66*). Conversely, several Marinimicrobia clades have been shown to participate in the biogeochemical cycling of sulfur and nitrogen (*67*). Abundant eukaryotic lineages for which the vulnerable fraction was above 50% included Dinophyceae, Bacillariophyta, and Ciliophora, which are all key planktonic groups in the ocean, considerably affecting global biogeochemical cycles. All MAST groups, some of which are heterotrophic and bacterivorous flagellates that interact with key photosynthetic picoplankton (*68*), are also predicted to be significantly affected. When resolving PC1 community lineages into MPGs (Fig. 4B), we predicted a large impact from temperature changes on Archaea, phototrophs, and phagotrophs, and in particular on gelatinous filter feeders. The critical role of gelatinous zooplankton within ocean trophic webs is increasingly being recognized as they may channel energy from picoplankton to higher trophic levels (*69*). The temperature sensitivity we predict for gelatinous filter feeders questions the paradigm that gelatinous zooplankton have been increasing in the past decades (*70*) and points toward the overall vulnerability of corresponding lineages to ocean warming in polar regions.

PC1 polar lineages predicted to be most sensitive to temperature were also identified at a lower taxonomic level (Fig. 4C) to infer potential species indicators of polar ecosystem change in response to ocean warming. Predicted bacterial genera as being most vulnerable to temperature change in polar regions were *Lentimonas* and *Methylotenera*, along with several uncharacterized OTUs (*n* = 30). *Lentimonas* spp. are specialized degraders of fucoidans and other complex polysaccharides (*71*). Their observed sensitivity to temperature variations may increase the recalcitrance of algal biomass to microbial degradation, which would affect the turnover of carbon sequestered in glycans that is vital for global carbon cycling (*72*). Methylotrophs of the family Methylophilaceae play a crucial role in the carbon cycle of aquatic habitats (*73*), and several *Methylotenera* spp. isolates are methylotrophic bacteria that can use a range of one-carbon compounds in coastal ocean ecosystems (*74*). Thus, these two genera appear to encompass rather specialist microbes with regard to their metabolism and are predicted to be affected by ocean warming in the polar ocean. Eukaryotic lineages predicted to be most sensitive to temperature included several abundant diatom genera: *Chaetoceros*, *Porosira*, *Proboscia*, and other genera belonging to Rhizosolenids and Mediophyceae. A single abundant genus of dinoflagellate was predicted to be affected by temperature change: *Protoperidinium*. For copepods, the genus *Pseudocalanus* and genera from the family Paracalanidae were found to be the most vulnerable. Picomonadida was the only heterotrophic protist family predicted to be vulnerable to temperature change.

Monitoring pelagic ecosystems under environmental stress due to ongoing climate change is challenging, but plankton species indicators may provide an accurate diagnosis of ecosystem health (*75*). Previous evidence suggests that the genera we predict as being most sensitive to temperature in polar ecosystems may be good candidates for plankton indicators of ocean warming. *Chaetoceros* constitutes a very large genus of marine planktonic diatoms and is a dominant component of phytoplankton communities contributing an estimated 20% of total oceanic primary production (*76*). *Chaetoceros* is abundant in polar oceans and is affected by temperature in laboratory experiments (*77*). A species distribution model previously showed that the annual median probability of occurrence of another diatom species *Rhizosolenia stolterfothii* was predicted to shift in the North Atlantic Ocean (*21*), suggesting that it, too, will be affected by anthropogenic climate change. Considering copepods, the abundance of the genus *Pseudocalanus* has continuously decreased within a decade (2003–2012) in East Greenland waters (*78*). Another line of evidence for the temperature sensitivity of the predicted genera comes from mesocosm experiments, in which the relative biomass of a diatom from the genus *Proboscia* (*Proboscia alata*) was negatively affected by temperature (*79*). As for dinoflagellates, a species of the genus *Protoperidinium* was shown to be less tolerant to prolonged temperature shifts in laboratory experiments (*80*).

Overall, these results underlie the differential responses of biome-specific plankton communities and associated functions to specific environmental changes. These findings provide new insights into community-specific environmental vulnerabilities of plankton lineages and associated functions. Plankton MPGs play central roles in the ecology and biogeochemistry of the polar (and global) oceans. Here, we predicted that specific plankton lineages and MPGs will be affected, which has substantial implications and may even worsen under currently projected scenarios of climate change (*33*) in the Arctic and in nutrient-rich oceanic regions.

This study provides a comprehensive cross-kingdom plankton interactome covering all major oceanic provinces, including the Arctic Ocean, a region that has lacked systematic standardized sampling. This global ocean ecological network constitutes an integrated resource to study plankton community structuring and prevalent planktonic associations across major marine biomes. Still, this resource is limited because predicted ecological associations do not demonstrate ecological interactions (*81*), and because it does not capture the dynamics of plankton interactions that are usually assessed using temporal or longitudinal samplings (*82*), nor the potential influence of horizontal gene transfer among plankton prokaryotes that may affect the stability of ecological interactions. Our knowledge of plankton microbiomes, symbioses, and host-parasite relationships remains limited (*83*). While planktonic interactions remain challenging to validate, our predictions are useful to further our understanding of ecosystem functioning and may constitute useful guidance for coculture experiments and a database for hypothesis testing. Today, high-throughput coculture experiments using microfluidics (*84*) and fabricated synthetic microbial ecosystems may help fill this gap (*85*).

Climate scenarios predict global changes in temperature, pH, and nutrient concentrations, which all greatly influence plankton physiology. Temperature can directly affect bacterial growth (*86*), grazing rates (*87*), and phytoplankton metabolism (*88*). Nitrogen availability is a primary limiting factor for marine phytoplankton (*89*). Ocean acidification caused by rising atmospheric CO_2_ can affect phytoplankton growth rates and is predicted to have a greater impact than warming or reduced nutrient supply on plankton ecological functions (*90*). Here, we identified and predicted distinct community vulnerabilities of the plankton interactome by studying its robustness to environmental perturbations. Overall, our findings imply differential effects of environmental change on biome-specific plankton communities resulting from biotic interactions and environmental stresses. While the influence of temperature is central, at the biome-specific community scale, salinity and nutrient concentrations were found to significantly influence plankton community structures as well. These associations support previous lines of evidence linking temperature and nutrient concentrations as the principal drivers of microbial plankton community variability (*91*).

These findings further advocate for the development of novel modeling paradigms targeting multiple biological scales (*92*) from genes to species and community levels (*93*). Our computational framework combining network analyses with niche modeling is generalizable and can be applied to various microbial ecosystems for assessing and predicting robustness to environmental perturbations. Here, specific lineage vulnerabilities were identified, but it remains an open question whether taxonomy, rather than function, is essential or not for predictive models given the potential functional redundancy in open microbial systems (*94*). Similar studies should be performed at the genomic level given that the molecular functions rather than the microbes themselves sustain marine biogeochemical processes (*95*).

## MATERIALS AND METHODS

### Data description

From 2009 to 2013, the *Tara* Oceans expedition collected samples at more than 200 stations across all significant oceanic provinces from oligotrophic to polar regions. Sampling stations were selected to represent distinct marine ecosystems at global scale, for which the sampling strategy and the methodology have been previously described (*96*). Sample provenance is described in table S1. Environmental data measured or inferred at the depth of sampling are available in table S2 and published at PANGAEA, Data Publisher for Earth and Environmental Science (www.pangaea.de). In this study, we limited our analyses to the euphotic zone, including only the samples from surface (SRF) and the Deep Chlorophyll Maximum (DCM). Two prokaryote-enriched size fractions (0.2 to 1.6 μm and 0.2 to 3 μm) were available and included in the analyses. For eukaryotes, the following size fractions were included (and consolidated as described below) in the analyses: “0.8 to 5 μm and 0.8 to 2000 μm,” “3 to 20 μm and 5 to 20 μm,” “20 to 180 μm,” and “180 to 2000 μm.” Because of these sampling constraints and the nonsystematic sequencing of all available samples, the *Tara* Oceans dataset is heterogeneous. Specifically, at polar stations, fractions 0.8 to 5 μm and 5 to 20 μm are less represented. Conversely, in nonpolar stations, sequencing data for the fraction 3 to 20 μm are nearly absent. To overcome this issue and increase sampling coverage, we considered that fractions 3 to 20 μm (for Arctic samples) and 5 to 20 μm (for non-Arctic samples) were equivalent, as well as fractions 0.8 to 5 μm and 0.8 to 2000 μm, as samples from these latter size fractions captured very similar diversity and community composition (fig. S11). When both size fractions were available for the same sampling site, the 0.8 to 5 μm size fraction was preferred. For the 3 to 20 μm/5 to 20 μm size fractions, only one station (TARA_124_SRF) was found to be in conflict, and we discarded the 3 to 20 μm sample. By doing so, we analyzed 115 sampling sites at which all considered size fractions were available.

### Data processing and taxonomic annotations

For the prokaryote-enriched size fraction (0.2 to 1.6 μm and 0.2 to 3 μm), taxonomic profiling was performed using 16*S* ribosomal gene fragments directly identified in Illumina-sequenced metagenomes (*4*). To profile taxonomic abundances from metagenomes using a reference-based method, two goals need to be achieved: (i) The reference database needs to be a balanced representation of the diversity space, and thus, the under-/overrepresentation of some taxa in the database needs to be corrected, which can be done by defining OTUs at a higher level (e.g., at genus level); (ii) the taxonomical units to be detected (if not defined in the database) need to be defined, which is the case in the SILVA database that is cataloguing reference sequences at the species and strain levels. Thus, we performed a preclustering of the SILVA database. By preclustering, OTUs at 97% similarity, OTUs are defined above the genus level, which also serves to balance the unequal representation of different taxa in the database. The 97% identity cutoff for the full 16*S* ribosomal RNA (rRNA) sequence was chosen as it matches the classical definition of 16*S* OTUs. Then, extracted 16*S* reads, named miTags, were mapped to cluster centroids of taxonomically annotated 16*S* rRNA gene reference sequences from the SILVA database (*59*) (release 128: SSU Ref NR 99), which had been clustered at 97% sequence identity beforehand using USEARCH v9.2.64. Additional methodological details are available in (*4*), and we used the OTU-level abundance matrix as provided by the authors. For the eukaryotic taxonomic profiling, we used metabarcoding data with the same methodology as in (*58*) to define OTUs. 18*S* rRNA gene V9 amplicons were clustered with the Swarm version 2.1.1 (with fastidious option, and *d* = 1) (*97*) and taxonomically and functionally annotated by global pairwise alignment (vsearch -usearch_global command) against an updated version (available at http://doi.org/10.5281/zenodo.3768951) of the PR2_V9 reference database (*3*). 18*S* rRNA gene V9 region polymerase chain reaction (PCR) primers also amplify some 16*S* rRNA gene V9; thus, we decided to filter out OTUs not assigned to eukaryotic reference sequences with more than 80% of identity ( “Bacteria”: 35,448 OTUs, “unassigned”: 31,406 OTUs, “Archaea”: 1806 OTUs, “root”: 58 OTUs, and “Organelle”: 583 OTUs). For the prokaryotic abundance matrix, we filtered out miTags assigned to “Eukaryota” (5283 OTUs), Chloroplast (468 OTUs), and Mitochondria (74 OTUs). After this filtering, we worked with six distinct matrices corresponding to each size fraction considered (see the Supplementary Materials).

On the basis of these taxonomic affiliations, we classified all taxa into MPGs as in (*3*). For prokaryotes, photosynthetic bacteria (i.e., cyanobacteria) were distinguished from heterotrophic/chemotrophic bacteria and archaea. For protists, the functional annotations of PR2_V9 (http://doi.org/10.5281/zenodo.3768951) were used. It encompasses a wide variety of protist taxa that are assigned to major functional groups: photosynthetic/mixotrophic protists, endophotosymbionts, hosts with endophotosymbionts (photohosts), parasitic protists, and free-living heterotrophs or phagotrophs (heterotrophic protists). For the mesozooplankton, the categories used corresponded to the most abundant taxonomic groups (such as copepods and chaetognaths) or feeding strategies.

Shannon diversity indices were calculated for each sample and provided by (*3*).

### Data transformation and filtering

All OTU abundance matrices were transformed using the centered log-ratio (CLR) transformation (*98*), while environmental parameters were standardized (or z-transformed), thus ensuring the normal distribution of the data. This is important, as FW (FlashWeave) was run in sensitive mode (see below) and thus computes partial correlation tests with Fisher’s z-transformation, which assume features to be multivariate Gaussian distributed in CLR-transformed space. The CLR transformation is widely used in microbiome data analysis, especially in association network reconstruction (*99*), as it copes with the compositional nature of microbiome data. As log transformation cannot be applied to zero values, we added beforehand a pseudo count of one to all elements of the matrix. Last, to reduce the high dimensionality of our data, which may be the source of false-positive predicted associations, we filtered each abundance matrix using a top-quartile filtering approach. For each sample, the upper quartile (Q3) of its nonzero abundance values was computed. An OTU was retained when its observed abundance was higher than Q3 in at least five samples.

### Network inference and stability procedure

The network inference was performed using FW v0.13.1 with default parameters (*38*). FW relies on the local-to-global learning framework and infers direct associations by searching for conditional dependencies between OTUs. Several heuristics are then applied to connect these “local” dependencies and infer a network. FW is significantly faster than other methods while achieving better or similar results and gives the possibility to include metavariables (such as the temperature). Although the latter feature seemed appealing, very few OTU-environmental factor associations were detected, which advocates for developing a complementary approach to study the environmental influence (see the section, “Network-based robustness analyses”). While FW includes a heterogeneous mode (FlashWeaveHE) and the *Tara* dataset is heterogenous itself, the low number of samples prevented its use. Thus, we used FW in “sensitive” mode without its embedded normalization because it was performed upstream to comply to our network inference strategy designed to deal with the multiple size fractions context described below.

We reconstructed graphs for each size fraction separately, running FW on the corresponding CLR-transformed abundance matrix. This first step only allows to discover intrafraction edges. To connect together the five resulting graphs and thus infer interfraction edges, we considered all 10 combinations of two size fractions and ran FW on the according concatenated matrices. This results in a metagraph, with OTUs from different size fractions being connected together.

To assess the robustness of intra- and interfraction edges and reduce the number of putative false-positive associations, we implemented a stability procedure inspired by the STARS model selection approach (*100*). As we did for two size fractions matrices, we built every combination of three size fractions matrices and obtained 10 three-fractions graphs. We then evaluated the stability of every metagraph edge by computing its frequency in the three-fractions graphs. This procedure computes a relative stability metric reflecting a given edge robustness to variation in both the number of samples and the number of OTUs. Edges with relative stability below 50% were removed from the metagraph.

### Estimation of FDR

Three null models were generated using two R packages (picante v1.7 and HMP v1.6). The HMP library provides the Dirichlet.multinomial function, which allows data matrix generation of OTUs following a Dirichlet distribution. Picante comes with a randomizeMatrix function and several methods to randomize the matrix. We used the frequency (that maintains OTU occurrence frequency) and trialswap (maintaining OTU occurrence frequency and sample OTU richness) approaches. Then, networks were inferred from these matrices using FW and the same procedure as for the observed matrices. We then estimated an FDR by comparing common edges between the observed and simulated networks. The highest FDR we obtained was 3.6% (with a number of iterations set to 10^8^) using the trialswap method.

### Literature-based validation of predicted interactions

To compare the performance and sensitivity of FW to similar co-occurrence network inference methods such as SPIEC-EASI (*99*), we estimated the graph accuracy by comparing edges with known (marine) biotic interactions. We limited our comparisons to Polar networks and compared edges with known interactions from the PIDA (*83*) (https://github.com/ramalok/PIDA) and GLOBI (*101*) databases (www.globalbioticinteractions.org/). We used the National Center for Biotechnology Information (NCBI) taxonomy for prokaryotes and PR2 taxonomy for eukaryotes to identify superkingdom, family, genus, and species levels. Then, we searched for known interactions from these databases in the networks by detecting all combinations of OTUs at the four taxonomic levels considered (fig. S12). Conserved associations across taxonomy ranks were estimated as follows. First, taxonomic ranks were extracted from NCBI Taxonomy database for prokaryotes and from PR2 database for eukaryotes. Next, for each pair of ranks, we counted the number of edges between nodes of each rank. Next, we repeated the procedure but now applied to the subnetwork induced by considering only nodes from a particular biome. Last, we calculated the proportion of edges for each rank pair in each biome with respect to the total network.

### Station-specific network extraction

To further explore the association between plankton community structures and abiotic factors, we extracted sampling station-specific subnetworks corresponding to local GPI interactomes containing only nodes of OTUs detected at a given sampling station. This procedure enabled the computation of graph topological metrics (mean degree, edge density, mean weight, mean strength, and transitivity) for each sampling station and enabled us to directly associate environmental parameters to local community structures.

### Marine biome assignations to OTUs

In the *Tara* Oceans dataset, each sample is associated with one specific marine biome (Coastal, Trades, Westerlies, or Polar). Using this information, we assigned each OTU to a biome or a combination of biomes according to its abundance profile. We did this by identifying biome(s) in which a given OTU is overrepresented, based on relative abundance, compared to other biomes using a Kruskal-Wallis (KW) test implemented in the Python package SciPy (version 1.2.1). Adjustments for multiple testing were performed using the Benjamini-Hochberg (BH) procedure implemented in statsmodels (version 0.9.0). For significant tests (FDR < 0.05), a post hoc Dunn’s test implemented in scikit-posthocs (version 0.6.1) was performed to determine in which biome(s) a given OTU was significantly overrepresented (FDR < 0.05). To determine the direction of the over-representation, we compared the mean values to identify and discard the “lower mean biome(s)” from the list of the OTU-associated biomes. In the GPI, we were able to assign biome(s) to a significant fraction of OTUs (41.1%). Numerical and categorical assortativities were determined with the corresponding functions from networkx 2.3.

### Network community detection and biome assignation to communities

We detected five communities in the GPI using an eigenvector-based network community detection algorithm (*102*) implemented in the networkx 2.3 python package. To assign biomes to these communities, OTU abundance tables were CLR-transformed and aggregated by community for each size fraction. CLR values for each community were grouped by biome, and a KW test was run to verify mean differences of communities among biomes (KW test column in table S4). As all *P* values were significant while controlling the FDR using the BH procedure, post hoc Dunn tests were performed to detect community pairwise differences between biomes (Dunn test *P* value columns in table S4). Biomes that were found significantly lower via the Dunn test were discarded from the biome assignation (Dunn test *z* score column in table S4). The five GPI communities were found prevalent in the Polar (*n* = 2), Westerlies (*n* = 1), or Trades (*n* = 2) biomes.

### Environmental optimum and tolerance range inference

Environmental optimum and tolerance range were calculated with the robust optimum method described in (*39*). For each OTU and a selection of environmental parameters, we determined the ecological optimum reflecting the optimal OTU living conditions relative to a given environmental parameter and a tolerance range around this optimum defined by lower and upper bounds. Here, Total Sum Scaling (i.e., read count divided by the total number of reads in each sample) normalization was applied to raw matrices to weight these optima and ranges by relative OTU abundances across sampling stations. For each OTU, the proportion of observed counts in a given sample is computed relatively to all samples. We use these proportions to fill a weighted vector of a fixed size (*n* = 10,000) with environmental values accordingly (i.e., if the proportion of observed counts for OTU1 in sample 1 represents 5% of the OTU1 abundance across all samples, then the weighted vector will be filled at 5% with the environmental value measured for sample 1). The ecological optimum is then defined as the median value (Q2) of this vector, the lower and upper limits as the first (Q1) and third quartile (Q3), respectively, and the tolerance (niche) range is given by the interquartile range (Q3 to Q1). Some environmental parameter values are missing [nonavailable (NA)] for some samples. To avoid inferring spurious ecological optima and tolerance ranges for OTUs for which many values are missing, we set a minimum threshold of 10 OTU observations with non-NAs and overall with a minimum of 30% non-NA values for it to be computed.

### General keystone index

The generalized keystone index (*59*) combines several centrality metrics in a single measure, which can then be used to rank nodes, revealing their topological importance in the network. Degree, betweenness, closeness, and subgraph centralities have been calculated using the Python library networkx (version 2.3), capturing the relevance of each node at different topological scales. Factor analysis was performed with the Python library sklearn (version 0.20.3) on those centralities to get the generalized keystone index associated with each node.

### Network-based robustness analyses

To simulate the effects of environmental change and predict their impact on the stability of plankton community structures, we designed a network attack procedure mimicking the potential effect of each environmental parameter’s variations onto the GPI. We progressively removed network nodes by bins (*n* = 200 nodes until the 10,000th node and then *n* = 1000 nodes) corresponding to environmental ranges, ordered from the smallest to the largest tolerance ranges for each parameter (within a given range, the nodes are randomly sorted). At each step, we computed the graph natural connectivity (*103*), a graph robustness metric, for the global interactome and for subgraphs corresponding to communities extracted from the GPI (see the “Network community detection and biome assignation to communities” section). By doing so, we could evaluate the vulnerability (or loss of robustness/stability) of the GPI at the global and community levels and detect OTUs and lineages that were actually targeted/affected first in the process.

Temperature and nutrient concentration changes are generally not independent; temperature increases metabolic rates, which may, in turn, increase nutrient uptake and cycling through the food web. Thus, both parameters may show a synergistic effect on plankton community structure (*104*). The potential for this abiotic synergy points toward a limitation of our in silico perturbation experiments because we did not integrate per se the whole set of environmental parameters that are necessary to properly define the ecological niche of a given OTU—nor the synergistic interactions between them. While an equal combination of different environmental tolerances can be assumed to define a niche (i.e., the cardinal product of limiting abiotic factors), we argue that this would bias our predictions because plankton species are differentially adapted and respond to environmental conditions. Lineage-specific adaptation may explain the differential sensitivity in the Polar biome, where temperature is significantly lower compared to nonpolar regions, and in the Westerlies biome, where nutrients are usually not limiting factors as compared to the Trades biome.

### Predicting most vulnerable community lineages and MPGs

To predict community-specific groups (marine plankton lineages and MPGs) most vulnerable to environmental change, we focused on most “abundant” OTUs, for which the total mean abundance was above 0.001. This cutoff corresponds to the mean relative abundance of all groups. The proportion of affected groups was computed as the factor between the total mean abundance and the affected mean abundance of a given group. For computing these affected proportions, we limited ourselves to environmental ranges corresponding to global mean anomalies projected for the end of the century by the CMIP6 model scenario SSP2-4.5 (*33*). Thus, environmental ranges considered here were 2.1°C for temperature, 0.5 PSS-78 for salinity, 0.7 μM for NO_2_, and 1.0 μM for PO_4_.

### Statistical analyses

Spearman correlations, followed by BH procedure (FDR < 0.01), were performed to test associations between network topology metrics and environmental parameters (Fig. 2A). KW tests followed by post hoc Dunn’s tests were performed using R (version 3.2.2) to determine significant differences across biome-specific interactome topological metrics (Fig. 2C). A Pearson’s chi-square test was performed to detect taxa associations enriched in each interactome community (fig. S3). Here, only pairs of taxa that co-occur in at least three communities and occur at minimum 50 times in total were tested. For these pairs, we performed a post hoc analysis for Pearson’s chi-square test on the residuals using the chisq.posthoc.test R package (https://chisq-posthoc-test.ebbert.nrw/) to identify within each community taxa pairs with a number of associations significantly diverging from random expectation. Wilcoxon’s rank sum tests were performed to compare distributions of natural connectivity for network environmental perturbations versus random perturbations (Fig. 3).

## SUPPLEMENTARY MATERIALS

Supplementary material for this article is available at http://advances.sciencemag.org/cgi/content/full/7/35/eabg1921/DC1

## Appendix

View/request a protocol for this paper from *Bio-protocol*.
